# Events preceding death among chikungunya virus infected patients: a systematic review.

**DOI:** 10.1590/0037-8682-0431-2019

**Published:** 2020-05-11

**Authors:** José Cerbino-Neto, Emersom Cicilini Mesquita, Rodrigo Teixeira Amancio, Pedro Emmanuel Alvarenga Americano do Brasil

**Affiliations:** 1 Instituto Nacional de Infectologia Evandro Chagas, Fundação Oswaldo Cruz, Rio de Janeiro, RJ, Brasil.; 2 Faculdade de Medicina, Universidade Estácio de Sá, Rio de Janeiro, RJ, Brasil.; 3 Hospital Federal dos Servidores do Estado, Rio de Janeiro, RJ, Brasil.

**Keywords:** Chikungunya Fever, Chikungunya virus, Death, Mortality, Disease Progression

## Abstract

Since its re-emergence in the late 1990s, there have been reports of Chikungunya fever (CHIK-F) presenting with severe or atypical findings. There is little knowledge regarding the clinical events leading to the death of patients with CHIK-F. This study aimed to systematically review the literature regarding CHIK-F and identify clinical features preceding death. We searched PubMed, Scopus, Embase, Lilacs, and IsiWeb for case-reports, case-series, or cohorts of CHIK-F reporting at least one death, up to December 2019. Fifty-seven reports were analyzed, including 2140 deaths. Data about specific clinical events that precede death are scarce. The central tendency of time between disease onset and death ranged from 2 days to 150 days. The most common clinical findings among decedents were fever (22.0%), arthralgia (15.7%), myalgia (10.7%), and headache (8.2%). Excluding pediatric populations, the reported central tendency of age among the decedents was 53 or older, with a non-weighted median of 67, ranging up to 80 years old. Authors mentioned organic dysfunction in 91.2% reports. Among all the 2140 decedents, the most common dysfunctions were cardiovascular (7.2%), respiratory (6.4%), neurological (5.4%), renal (4.2%), liver (3.0%), and hematological (1.3%) dysfunction. Exacerbation of previous diabetes (5.6%) or hypertension (6.9%) was mentioned as conditions preceding death. Currently, older age, primary neurological, cardiovascular, or respiratory dysfunction and a previous diagnosis of diabetes or hypertension are the main clinical events preceding death.

## INTRODUCTION

Chikungunya virus (CHIK-V) was first identified in 1952, in Tanzania^1^. Later, since the 1960s, outbreaks have occurred in Asia and Africa^1^. Chikungunya fever (CHIK-F) was seldomly reported during the 20^th^ century. However, in 1999, there was an outbreak in the Democratic Republic of Congo, and in 2005 outbreaks occurred in the Indian ocean islands subsequently spreading to some Asian countries, Europe, and the Americas^1^. Recently, it was added to the list of neglected tropical diseases^2^.

CHIK-F is an acute febrile illness commonly presenting with acute onset of pyrexia along with inflammatory arthralgia and arthritis, sometimes with severe pain, most frequently in the extremities (wrists, ankles, and phalanges)^3^. Diagnostic laboratory investigations include serological testing and polymerase chain reaction, sometimes performed in cerebral spinal fluid^3^. Some guidelines or reviews are indicating that CHIK-F may be asymptomatic in up to 13% of all cases, with rare atypical presentations occurring in approximately 1% of all cases^3^ ^,^ ^4^. 

CHIK-F lethality is believed to be as low as 0,1%, but the disease can be easily mistaken with Dengue fever or other acute febrile illness^5^ ^,^ ^6^, and its mortality may be underestimated during outbreaks^6^. Clinical alarms that would justify hospitalization are sometimes unclear in guidelines but may include severe manifestations or atypical findings, such as intense pain, bleeding, dehydration, thrombosis or decompensation of previous clinical conditions^3^ ^,^ ^5^. Additionally, it is not clear from reference documents or current guidelines if clinical events preceding death are primarily attributable to CHIK-V, or if these events are exacerbations of previous conditions, or how often they occur. It is also unclear which CHIK-F patients are at higher risk of death. Therefore, this study aimed to systematically review the medical literature regarding CHIK-F deaths and identify clinical features that precede death among CHIK-F patients. This will uncover features that could be used severity alerts, guiding the need for hospitalization or intensive care. 

## METHODS

This review’s protocol was registered at the international prospective register of systematic reviews (Prospero) and can be found at http://www.crd.york.ac.uk/PROSPERO/display_record.php?ID=CRD42017056692.

The population of interest for this review was defined as any case of laboratory-confirmed CHIK-V infection, for example by serological or DNA amplification methods. Additionally, the report contained at least one death attributable to CHIK-F. Any observational or interventional study design was accepted. There was no period, language, or publication status restriction, and symposium, congress summary, or posters were all accepted. Reports with data regarding exclusively pregnant patients or CHIK-V vertical transmission were of no interest to this review.

The search was based on two strategies. First, the search of remote electronic databases, followed by the manual search of references at the bibliography of the full papers retrieved.

The remote database searches were performed using PubMed, LILACS, Scopus, Embase, and Web of Science. The first searches were performed on February 23^rd^, 2017 and the last updated search was done on December 18^th^, 2019. The search strategies were as follow: PubMed - chikungunya AND (death* OR dead OR died OR mortality OR lethality OR fatal*) AND (severe OR "intensive care" OR ICU OR clinic* OR myocardi* OR hepatitis OR enceplhalopath* OR meningit* OR sepsis OR septic); Web of Science - TS=(chikungunya) AND TS=(death* OR dead OR died OR mortality OR lethality OR fatal*) AND TS=(severe OR "intensive care" OR ICU OR clinic* OR myocardi* OR hepatitis OR enceplhalopath* OR meningit* OR sepsis OR septic); Scopus - chikungunya AND (death* OR dead OR died OR mortality OR lethality OR fatal*) AND (severe OR "intensive care" OR ICU OR clinic* OR myocardi* OR hepatitis OR enceplhalopath* OR meningit* OR sepsis OR septic); Embase - chikungunya AND (death* OR dead OR died OR mortality OR lethality OR fatal*) AND (severe OR "intensive care" OR ICU OR clinic* OR myocardi* OR hepatitis OR enceplhalopath* OR meningit* OR sepsis OR septic); Lilacs - chikungunya AND (morte* OR mortalidade OR letalidade OR morre* OR death* OR dead OR died OR mortality OR lethality OR fatal*) AND (sever* OR "intensive care" OR "terapia intensiva" OR ICU OR UTI OR clinic* OR myocardi* OR miocardio* OR hepatitis OR hepatite OR enceplhalopath* OR encefalopatia OR meningit* OR sepsis OR septic).

All four authors reviewed the abstracts and retrieved data from the full text. Each reviewer performed the classification and data extraction independently. The abstracts were downloaded to Zotero® reference manager. After the first screening, full reports files were captured, and then the extracted data were recorded using the RedCap® software. At each round of classification, a third reviewer voted on how to solve disagreements. 

For data collection, a specific form was designed and piloted several times until an agreement was reached by the reviewers. The forms contained study characteristics such as publication type, population characteristics such as the country where research occurred, special characteristics such as critically ill or with a neurologic manifestation, period of inclusion, comorbidities, and organic dysfunction. Regarding organic dysfunction, the following definitions were adapted from the SOFA score (Sepsis-related Organ Failure)^7^: bilirubin > 1.2mg/dl or changes in INR due to liver dysfunction; the need for orotracheal tube or non-invasive ventilation for respiratory dysfunction; the need for blood components transfusion or platelets count < 150.000/ mm^3^ for hematologic dysfunction; the need for vasoactive agents, or mean arterial pressure < 70 or shock for cardiovascular dysfunction; the need for dialysis or creatinine > 1.2mg/dL for renal dysfunction; seizures, abnormal behavior, abnormal motor functions, abnormal sensorial functions, or Glasgow score < 15 for neurologic dysfunction. 

The critical appraisal for all reports included was performed using the checklist for case series of the Joanna Briggs Institute - University of Adelaide available at (http://joannabriggs.org/research/critical-appraisal-tools.html)^8^. The analysis plan is based on narrative and descriptive analysis, and frequencies of events of interest. The main subgroups of interest are the elderly and those with comorbidities. The critical appraisal was used only for overall quality evaluation. 

## RESULTS

**FIGURE 1:**
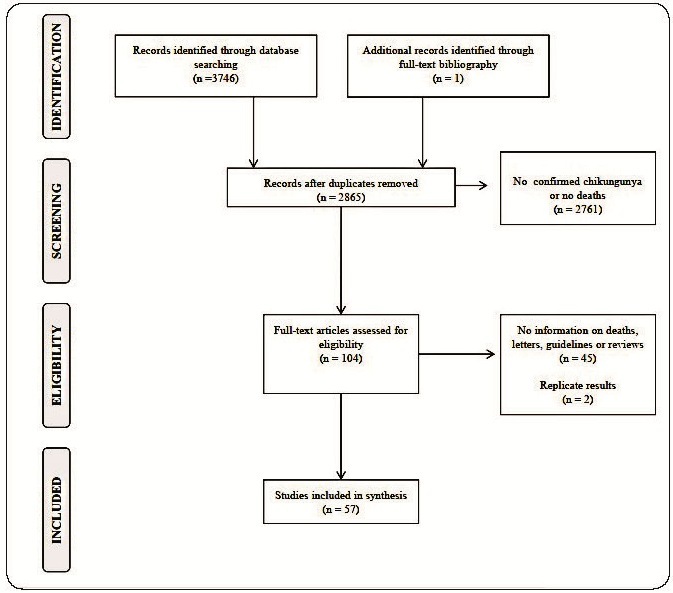
Inclusion and exclusion flowchart.

After performing the remote database search, 3746 abstracts were retrieved. After the removal of replicated abstracts and applying the eligibility criteria, a total of 57 reports were analyzed^9^ ^-^ ^64^. (Figure 1) The majority of exclusions were due to the absence of death reports in the original study sample. The included reports were mostly case series (45.6%) followed by case reports (28.1%), and observational follow-up studies (26.3%). (Table 1) The review included 2140 deaths. (Table 1) The overall death rate ranged from 0.1% to 100%, however, considering only the observational follow-up studies, the median non-weighted death rate was 3.1%, ranging from 0.1% to 36.7% in the general population, and 36.7% ranging from 27.7% to 48.8% among critically ill subjects. 

**TABLE 1:** Study description and Chikungunya death rates.

Study	Study type	Country	Period	Special population	Days to death (central tendency)	Number of Deaths	Chikungunya cases	Death rate (%)
Sarkar - 1967	Observational with follow-up	India	1963-1965	General population	-	2	64	3.1
Ramful - 2007	Case series	France	2005-2006	Neonatal	-	1	38	2.6
Rampal - 2007	Case series	India	2006-2006	Neurologic	28	6	20	30
Renault - 2007	Case series	France	2005-2006	General population	-	203	16050	1.3
Ernould - 2008	Case series	France	2006-2006	Pediatric	-	2	65	3.1
Ganesan - 2008	Case report	India	2008-2008	Neurologic	-	1	2	50
Lemant - 2008	Case series	France	2005-2006	Critically ill	5	16	33	48.5
Robin - 2008	Case series	France	2006-2006	Pediatric with neurological	5	2	30	6.7
Economopoulou - 2009	Case series	France	2005-2006	Atypical manifestations	-	65	878	7.4
Suryawanshi - 2009	Case series	India	2006-2006	General population	-	3	87	3.4
Tandale - 2009	Observational with follow-up	India	2006-2006	Hospitalized	-	26	90	28.9
Tournebize - 2009	Case series	France	2005-2006	Neurologic	-	2	26	7.7
Chua - 2010	Case report	Malaysia	2010-2010	Pediatric	5	1	1	100
Kee - 2010	Case report	Singapure	2008-2008	Immunocompromised	150	1	2	50
Sam - 2010	Case report	Malaysia	2008-2008	Cardiologic	5	1	1	100
Gaüzère - 2011	Observational with follow-up	France	2005-2006	Critically ill	-	21	43	48.8
Gurav - 2012	Case series	India	2011-2011	General population	-	3	179	1.7
Hertz - 2012	Observational with follow-up	Tanzania	2007-2008	Hospitalized	-	5	55	9.1
Garcia - 2014	Case report	Philippines	2014-2014	General population	-	2	2	100
Bhooshan - 2015	Observational with follow-up	India	2010-2010	Hospitalized	-	5	79	6.3
Hoz - 2015	Case report	Colombia	2014-2014	General population	5	3	3	100
Shaikh - 2015	Case report	India	2014-2014	Neurologic	8	1	1	100
Sharp - 2015	Case series	Puerto Rico	2014-2014	Post mortem	6	28	28	100
Thiery - 2015	Observational with follow-up	France	2014-2014	Critically ill	-	18	65	27.7
Torres - 2015	Case series	Venezuela	2014-2015	Critically ill	6	3	4	75
Viasus - 2015	Case report	Colombia	2015-2015	Geriatric	5	1	1	100
Betancur - 2016	Case report	Colombia	NA-NA	Critically ill	8	1	1	100
Carta - 2016	Observational with follow-up	Venezuela	NA-NA	General population	-	3	287	1
Crosby - 2016	Case series	France	2014-2014	Critically ill	-	18	65	27.7
Gérardin - 2016	Observational with follow-up	France	2005-2009	Neurologic	-	7	57	12.3
López - 2016	Observational with follow-up	Puerto Rico	2014-2014	General population	-	2	1469	0.1
Méndez-Domínguez - 2016	Case report	Mexico	2015-2015	Pediatric	4	1	1	100
Mercado - 2016	Case series	Colombia	2014-2015	Dengue co-infection	3	7	7	100
Perti - 2016	Observational with follow-up	USA	2014-2014	General population	-	6	180	3.3
Rollé - 2016	Observational with follow-up	France	2014-2014	General population	-	14	110	12.7
Rosso - 2016	Case report	Colombia	2014-2014	Dengue co-infection	8.5	1	1	100
Balavoine - 2017	Case series	France	2014-2015	Neurologic	109	2	13	15.4
Epelboin - 2017	Case report	France	2014-2014	General population	6	1	1	100
Epelboin - 2017	Case report	Guiana	2014-2014	General population	10	1	1	100
Evans-Gilbert - 2017	Case report	Jamaica	2014-2014	Neonatal	4	2	2	100
Godaert - 2017	Case series	France	2014-2014	Geriatric	58	35	385	9.1
Sá - 2017	Case series	Brazil	2016-2016	General population	15	4	4	100
Cardona-Ospina - 2018	Case report	Colombia	2018-2018	Dengue/Leptospirosis co-infection	-	1	1	100
Colavita - 2018	Case report	Italy	2017-2017	General population	3	1	1	100
Dorleans - 2018	Case series	France	2013-2015	Hospitalized	-	74	1836	4
Gupta - 2018	Observational with follow-up	India	2016-2016	Critically ill	-	22	60	36.7
Koeltz - 2018	Case series	France	2014-2015	Critically ill	-	18	64	28.1
Melo - 2018	Case series	Brazil	NA-NA	Post-mortem	-	22	60563	0
Mercado - 2018	Case series	Colombia	2014-2015	Post-mortem	2	13	13	100
Silva Junior - 2018	Case series	Brazil	2016-2017	Chronic kidney disease	-	383	177931	0.2
Gohel - 2019	Observational with follow-up	India	2016-2016	Neurologic	-	3	110	2.7
Lima Neto - 2019	Case series	Brazil	2016-2017	General population	15	169	80000	0.2
Pinto - 2019	Case series	Brazil	2016-2017	Chronic kidney disease	-	269	94666	0.3
Rahman - 2019	Case series	Bangladesh	2017-2017	General population	-	3	690	0.4
Ray - 2019	Observational with follow-up	India	2016-2016	General population	-	7	21	33.3
Silva Junior - 2019	Observational with follow-up	Brazil	2016-2017	General population	-	383	182731	0.2
Simião - 2019	Case series	Brazil	2016-2018	General population	15	245	46495	0.5

**NA:** not assigned; **“-“:** not informed.

France (17), India (11), Brazil (7), and Colombia (7) were the countries with the most report. Of note, the majority of French reports came from the French ultramarine territories. (Table 1) The reported central tendency of time between disease onset and death ranged from 2 days to 150 days, with a non-weighted median of 6 days. (Table 1) None of the follow-up studies reported the time between disease onset and death. It seems that the time to death among the geriatric populations was usually higher than that of the general population and other special populations such as in the critically ill, pediatric, or in patients with neurological symptoms. 

There were some reports of deaths among children under 12 years old, however, the central tendency of age among the decedents was 53 years old or higher, with a non-weighted median of 67, ranging up to 80 years old. The deaths seem to be equivalently distributed between males and females. Regarding the diagnostic tests, polymerase chain reaction (PCR) in blood samples was more common, reported 27 times, while serology was reported 16 times. In six reports, both blood tests were used. Other non-ordinary tests were also mentioned, mainly PCR and serological tests performed using cerebral-spinal fluid (CSF) samples. These were usually conducted in critically ill patients. 

Information on clinical manifestations of fatal cases is very scarce. Fever, rash, and arthralgia are mentioned in approximately half of the reports, but the presence or absence of other clinical manifestations is ignored in 70.0% to 85.0% of the reports depending on the clinical finding. Among all the 2140 decedents, the most common clinical findings were fever (22.0%), arthralgia (15.7%), myalgia (10.7%), headache (8.2%), skin rash (5.7%), nausea/vomiting (5.3%), arthritis (4.3%), and bleeding (2.6%), while the remaining clinical findings were present in less than 1.0% of all decedents. 

The authors mentioned organic dysfunction in 91.2% of the reports. Neurologic dysfunctions were the most mentioned (47.4%), followed by respiratory (43.9%) and cardiovascular (42.1%) dysfunctions. Hepatic, hematological, and renal dysfunctions were also mentioned in up to 31% of the reports. Neurological findings, when reported, were present in 9% to 100% of the deceased. (Table 2) Among all the 2140 decedents, assuming that dysfunctions were not present when not reported, the most common dysfunctions were cardiovascular (7.2%), respiratory (6.4%), neurological (5.4%), renal (4.2%), liver (3.0%), and hematological (1.3%) (Table 2). 

**TABLE 2:** Number of reported clinical dysfunctions among the dead subjects.

Study	Number of deaths	Liver (%)	Respiratory (%)	Hematological (%)	Cardiovascular (%)	Renal (%)	Neurological (%)
Sarkar - 1967	2	-	-	-	-	-	-
Ramful - 2007	1	-	-	1(100.0)	-	-	-
Rampal - 2007	6	-	1(16.7)	-	-	-	6(100.0)
Renault - 2007	203	-	-	-	-	-	-
Ernould - 2008	2	-	-	-	1(50.0)	-	1(50.0)
Ganesan - 2008	1	-	-	-	-	-	1(100.0)
Lemant - 2008	16	2(12.5)	4(25.0)	-	3(18.8)	-	4(25.0)
Robin - 2008	2	-	-	-	2(100.0)	-	2(100.0)
Economopoulou - 2009	65	7(10.8)	18(27.7)	-	22(33.8)	3(4.6)	6(9.2)
Suryawanshi - 2009	3	-	-	1(33.3)	-	-	2(66.7)
Tandale - 2009	26	5(19.2)	5(19.2)	1(3.8)	5(19.2)	15(57.7)	20(76.9)
Tournebize - 2009	2	-	-	-	-	-	2(100.0)
Chua - 2010	1	1(100.0)	1(100.0)	1(100.0)	1(100.0)	1(100.0)	-
Kee - 2010	1	-	1(100.0)	1(100.0)	-	1(100.0)	-
Sam - 2010	1	-	1(100.0)	1(100.0)	1(100.0)	1(100.0)	1(100.0)
Gaüzère - 2011	21	3(14.3)	7(33.3)	-	7(33.3)	1(4.8)	3(14.3)
Gurav - 2012	3	-	-	-	-	-	-
Hertz - 2012	5	-	-	-	-	-	1(20.0)
Garcia - 2014	2	-	1(50.0)	-	-	2(100.0)	-
Hoz - 2015	3	1(33.3)	3(100.0)	2(66.7)	2(66.7)	3(100.0)	1(33.3)
Shaikh - 2015	1	-	-	-	-	-	1(100.0)
Sharp - 2015	28	-	-	-	-	-	-
Thiery - 2015	18	-	-	-	-	-	-
Torres - 2015	3	3(100.0)	3(100.0)	2(66.7)	3(100.0)	3(100.0)	-
Viasus - 2015	1	-	1(100.0)	1(100.0)	1(100.0)	1(100.0)	-
Betancur - 2016	1	-	1(100.0)	1(100.0)	1(100.0)	1(100.0)	-
Carta - 2016	3	-	-	-	-	-	-
Crosby - 2016	18	-	-	-	-	-	-
Gérardin - 2016	7	-	1(14.3)	-	5(71.4)	-	7(100.0)
López - 2016	2	-	-	-	-	-	-
Méndez-Domínguez - 2016	1	1(100.0)	1(100.0)	1(100.0)	1(100.0)	1(100.0)	1(100.0)
Mercado - 2016	7	-	-	5(71.4)	1(14.3)	3(42.9)	-
Perti - 2016	6	2(33.3)	1(16.7)	5(83.3)	2(33.3)	3(50.0)	4(66.7)
Rollé - 2016	14	-	-	-	12(85.7)	-	-
Rosso - 2016	1	1(100.0)	1(100.0)	1(100.0)	1(100.0)	1(100.0)	1(100.0)
Balavoine - 2017	2	-	2(100.0)	-	-	-	2(100.0)
Epelboin - 2017	1	-	-	1(100.0)	-	-	1(100.0)
Evans-Gilbert - 2017	2	-	1(50.0)	1(50.0)	1(50.0)	1(50.0)	1(50.0)
Godaert - 2017	35	-	-	-	-	-	-
Sá - 2017	4	-	4(100.0)	-	-	-	4(100.0)
Gupta - 2018	22	-	-	-	-	-	-
Koeltz - 2018	18	-	-	-	2(11.1)	-	-
Melo - 2018	22	-	-	-	-	-	-
Mercado - 2018	13	-	6(46.2)	-	8(61.5)	6(46.2)	-
Silva Junior - 2018	383	19(5.0)	-	-	-	-	-
Gohel - 2019	3	-	-	-	-	-	3(100.0)
Lima Neto - 2019	169	-	-	-	-	-	-
Pinto - 2019	269	-	-	-	-	-	-
Rahman - 2019	3	-	1(33.3)	-	-	-	2(66.7)
Ray - 2019	7	-	-	-	-	-	-
Silva Junior - 2019	383	-	-	-	-	-	-
Simião - 2019	245	-	29(11.8)	-	13(5.3)	-	-

**“-“:** not informed.

Comorbidities such as hypertension, rheumatic conditions, hepatic conditions, or diabetes were also seldomly mentioned. However, the authors mentioned the exacerbation of previous comorbidities in 64.3% of the reports. When present, exacerbation of these previous conditions preceding death ranged from 32% to 100% of deaths within each report. (Table 3) Hypertension (6.9%) and diabetes (5.6%) were the most common comorbidities; nevertheless, liver disease, COPD, chronic kidney disease, heart disease, and myasthenia gravis were also mentioned as clinical events preceding death. (Table 3) Among all the 2140 decedents, the most common comorbidities, were diabetes or hypertension (5.6% and 6.9% respectively), while rheumatic and hepatic conditions were reported in less than 1% of the deceased (Table 3). 

**TABLE 3:** Reports mentioning comorbidities (number and percentage) among the deceased subjects.

Study	Number	Death due	Hypertensio	Rheumati	Hepatopath	Diabete	Other
	of deaths	to comorbidit	n (%)	c (%)	y (%)	s (%)	(%)
		y (%)					
Renault - 2007	203	40.4	-	-	-	-	-
Lemant - 2008	16	50.0	-	-	1(6.2)	5(31.2)	COPD
Tandale - 2009	26	38.4	-	-	-	-	-
Tournebize - 2009	2	50.0	-	-	-	-	Myasthenia
Kee - 2010	1	100.0	-	-	-	-	-
Sam - 2010	1	100.0	1(100.0)	-	-	-	Cardiac Failure
Gaüzère - 2011	21	38.0	-	-	-	-	score IGSII
Gurav - 2012	3	100.0	1(33.3)	-	-	1(33.3)	Ischemic heart disease
Hertz - 2012	5	80.0	-	-	-	1(20.0)	Pleural effusion
Hoz - 2015	3	33.0	1(33.3)	-	-	-	Benign prostatic hyperplasia
Sharp - 2015	28	32.1	15(53.6)	-	-	11(39.3)	Obesity; Sickle cell anemia; Chronic kidney disease; Leptospirosis
Viasus - 2015	1	100.0	-	-	-	-	-
Betancur - 2016	1	100.0	-	1(100.0)	-	-	-
López - 2016	2	100.0	-	-	-	-	-
Perti - 2016	6	33.3	-	-	1(16.7)	-	-
Cardona-Ospina - 2018	1	100.0	1(100.0)	-	-	-	Chronic heart failure; Atrial fibrillation; Chronic venous disease; Right bundle branch block
Colavita - 2018	1	100.0	1(100.0)	-	-	-	Ischemic heart disease; Chronic inferior limbs arteriopathy; Bilateral carotid atherosclerosis

**“-“**: not informed; **IGSII**: *indice de gravité simplifié.*

**FIGURE 2:**
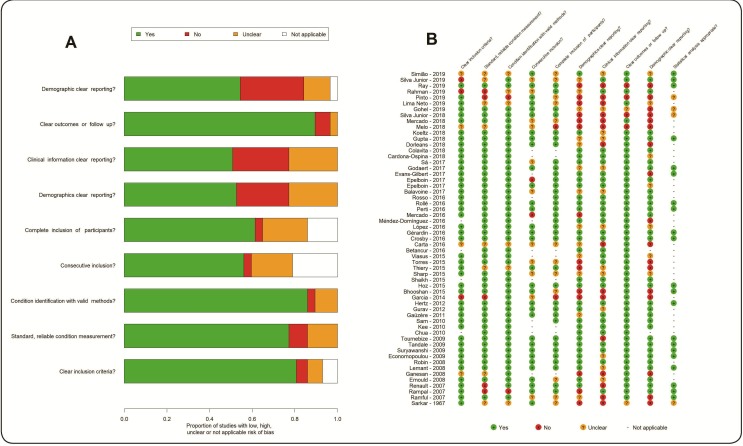
Original reports critical appraisal.

The critical appraisal showed an acceptable risk of bias. Of course, it is very difficult to critically assess different study designs for the same purpose. Nevertheless, the dimensions with the most questionable or not acceptable risk of bias were the dimensions “clinical information” and “demographics” of the death cases. (Figure 2A) This impression also appears in all tables as the absence of information. “Not applicable” also appears frequently in specific dimensions, for example, it does not make sense to evaluate consecutive inclusion or complete inclusion of a single case report. (Figure 2B) Often, systematic reviews make sensitive analyses using only the low risk of bias studies. However, the synthesis was intended to be only descriptive as there was a considerable high degree of lack of data from original studies. The sensitivity analysis did not lead to any additional interpretation. 

## DISCUSSION

The main results to be discussed are: (a) CHIK-F lethality in the general population is believed to be less than 1%; however it can be as high as 33% and, additionally, among the critically ill requiring life support, lethality can be as high as 48%; (b) data about the chain of clinical events that lead to death are scarce; (c) nevertheless, the currently available data suggest that age above 53 years old, primarily respiratory, cardiovascular, or neurological dysfunction, as well as decompensated comorbidities, are the main clinical events preceding death. 

This review was not designed to estimate mortality rates, and according to the inclusion criteria, only reports with deaths were analyzed. This may overestimate the mortality, as reports aiming to estimate mortality, but not observing death, would never be considered in the analyses. Nevertheless, the CHIK-F mortality estimation remains a topic of discussion. Although it is believed that CHIK-F has a low lethality, there is evidence that deaths attributable to CHIK-F are underreported^65^ ^-^ ^67^, and during CHIK-F epidemic periods, the excess of all-cause mortality in the general population may increase by 1%, or more than the expected mortality, especially in individuals belonging in the extreme age groups^68^ ^-^ ^74^. 

The CHIK-F intra-hospital lethality varies considerably, ranging from 4% to 29%^17^ ^,^ ^19^ ^,^ ^26^ ^,^ ^57^ ^,^ ^71^. Also, there is evidence pointing that the lethality in critically ill CHIK-F group of patients is higher than that in all critically ill patients in the intensive care units (ICU)^24^ ^,^ ^50^, although this may be controversial when adjusting lethality for severity scores^24^ ^,^ ^36^. When compared to CHIK-F overall lethality, specific groups, such as advanced age and decompensated comorbidities, may have increased lethality^17^, but again this is controversial when adjusting for the Acute Physiology and Chronic Health Evaluation (APACHE) II score (which age is one of its predictors)^49^. 

So far, there are very few studies adjusting for the effects of predictors of death. Nevertheless, there is a mix of data pointing to similar clinical features of disease severity observed in this review leading to hospitalization of CHIK-F cases. There is evidence pointing to a higher rate of hospitalization of CHIK-F patients when compared with the general population, and this is more evident in the extreme age groups^47^ ^,^ ^57^ ^,^ ^75^, and with diabetes or hypertension^36^ ^,^ ^57^. Additionally, there is a clear trend of disease severity proportion being higher in older patients^17^ ^,^ ^38^ ^,^ ^53^. Also, there is evidence that subjects with a typical presentation are less likely to be hospitalized, and subjects with signs of bleeding, vomiting, leukopenia, severe arthralgia, with neurological findings, or in the extreme age groups are more likely to be hospitalized^57^ ^,^ ^75^ ^,^ ^76^. Although comorbidities and unusual CHIK-F presentation are more frequent as age increases, there seems to be a small effect of age on hospitalization even when adjusted for comorbidities^76^. Additionally, diabetes-specific mortality also increased during CHIK-F epidemic^77^ as patients with CHIK-F and diabetes had a higher proportion of severe infection when compared with those without diabetes^19^. A similar interpretation is possible with systemic hypertension^17^.

The more frequent observation of severe cases in the extreme age groups can be explained by the lack of maturity in the immune system in newborns and a gradual deterioration of the immune system in old age. This phenomenon compromises the host’s ability to defend against infection^78^.

There is evidence that CHIK-F patients admitted to ICU are younger than patients admitted to the general ICU for other reasons. Nevertheless, the average severity score *indice de gravité simplifié* (IGS II) is higher among CHIK-F patients^24^. Additionally, there were no lethality differences among different age groups of critically ill CHIK-F patients when adjusting for the IGS II severity^24^, and the effect of age on lethality may be suppressed when adjusting for the APACHE II severity score^49^. However, older age may still be associated with death in hospitalized patients even when adjusting for comorbidities^19^ ^,^ ^63^. 

Predictors of death in a sample of elderly patients with up to seven days of fever at the time of hospital admission were estimated to be: sensorimotor deficit, confusion or delirium, concurrent cardiovascular disorders, concurrent respiratory infection, absence of musculoskeletal pain, history of alcoholism, and concurrent digestive symptoms. The strongest predictors of this multivariable analysis with this cohort in descending order are concurrent cardiovascular disorders, concurrent respiratory infection, and sensorimotor deficit^47^. The internal performance of this survival model was good. However, this research did not provide a tool for individual risk estimation and none of its results were validated in an independent sample. The main issue with this result is that it includes only the elderly, therefore, generalization to the overall population is limited as older age could not be assessed as a predictor. Another multivariable analysis conducted with secondary surveillance data at a Brazilian Northeastern state during an epidemic period included 383 deaths and identified the following predictors: older age (the strongest predictor), male sex, hypertension, diabetes, chronic kidney disease, leukopenia, and vomiting^63^. These predictors were very similar to the clinical features preceding death identified in this review. 

There are case reports on septic shock exclusively related to CHIK-V^42^ ^,^ ^66^ ^,^ ^79^, and case reports of very unusual presentations preceding death such as peritonitis and intestinal perforation, possibly also related to the use of Nonsteroidal Anti-Inflammatory Drugs (NSAIDs)^17^ ^,^ ^80^. Also, population common characteristics, such as alcohol abuse, are reported as associated to CHIK-F mortality^17^. 

Despite that, the incidence of unusual clinical manifestation was estimated to be high^76^ (24%), the incidence of neurological complications among CHIK-F patients is often estimated to be less than 1%^58^ ^,^ ^76^ ^,^ ^81^. Nevertheless, different from other clinical manifestations related to comorbidities, neurological findings seem to be directly attributed to CHIK-F infection^82^; therefore, the presence of a neurological finding could be a worrisome sign of potential lethality. Central nervous system disorders are much more frequent than peripheral manifestations^82^. The most common neurological manifestations in adults were estimated to be Guillain-Barré syndrome, 64.3%; meningoencephalitis, 24.1%; and acute demyelinating encephalomyelitis, 8.0%^81^ while in children seizure was the most common^83^. Of course, other less frequent manifestations such as transverse myelitis and optic neuritis may be observed as well^79^ ^,^ ^81^ ^,^ ^82^. It seems that the illness duration up to the time of hospital admission is longer for subjects with neurological signs^62^, and the time from infection to neurological manifestations and its lethality differ^81^. Guillain-Barré syndrome has the longest time to presentation and lowest lethality while meningoencephalitis has the shortest time to presentation and highest lethality^81^. Autopsy findings point to systemic involvement including encephalitis, hepatitis, myocarditis, and pulmonary edema^55^, and it is possible that many patients with a neurologic clinical features do not have compatible findings at neurological imaging^16^ ^,^ ^83^. Nevertheless, cases indicating the potential benefits of passive immunization when encephalitis is present have been reported^84^. 

The limitations of this review are mainly related to scarcity and heterogeneity of original data on deceased subjects. The review included case reports, case-series, and cohort studies that are inherently prone to different sources of errors and misinterpretations and have different kinds of inherent design limitations. For instance, it is very difficult to give a comprehensive interpretation of lethality from case-series and case reports as all the subjects of the same population at risk of death may not be observed or the population may not even be accurately defined and the number of deaths is so low that estimates may be unacceptably inaccurate. 

There are also limitations inherent to the interpretations of the causes of death. In this review, as there are very few studies in the literature estimating the effect of determinants of deaths among CHIK-F patients, the approach was similar to the death certificates approach. Once a death is reported, we attempted to identify the chain of clinical events leading to death. This is somehow different from the epidemiological approach of determinants or predictors where the risk of death given a condition is the main concern. It is very difficult to estimate death rates from case series or cohort studies among the general population because, at some point, subjects with disease progression will probably become hospitalized. Additionally, when a subject has several conditions, it is difficult to know which conditions are the main contributors to death, unless a study is designed for this purpose. For instance, when comorbidities are present, it is very difficult to accurately determine if CHIK-F presentation is severe because of presence or exacerbation of comorbidities, or alternatively if comorbidities decompensate due to severe CHIK-F. As there are several studies underreporting CHIK-F deaths, it may be that most physicians believe CHIK-F exacerbates underlying chronic conditions leading to death. This is particularly important while assessing CHIK-F mortality, as CHIK-V may often not be considered the main contributor to death. 

In conclusion, data about specific events known to increase the risk of death is scarce and heterogeneous. The same holds for clinical events preceding CHIK-F deaths. Currently, extremes of age, mainly the elderly, cardiovascular, neurological, or respiratory dysfunctions, and decompensated comorbidities are the main clinical events preceding death. Additionally, the data point to primary dysfunctions, as the main clinical events preceding deaths, to be more frequent than exacerbation of comorbidities. The evidence supporting exacerbation of comorbidities preceding deaths is stronger regarding when comorbid systemic hypertension and diabetes are present, although other comorbidities may be contributing. It would be highly valuable for guidelines and recommendations to explicitly define alarming features in CHIK-F that increase the risk of death. This will help in justifying hospitalization or close monitoring. Future research aimed at the development or validation of clinical prediction tools will be of great use. 
